# Representation of gender and people of color among healthcare professionals in medical comics – a document analysis

**DOI:** 10.3205/zma001726

**Published:** 2025-02-17

**Authors:** Cyrus Amin Parsa, Johanna Hirsch, Katrin Schüttpelz-Brauns

**Affiliations:** 1 Medical Faculty Mannheim of Heidelberg University, Division of Studies and Teaching Development, Department of Medical Education Research, Mannheim, Germany

**Keywords:** document analysis, gender role, medical comic, people of color, stereotyping

## Abstract

**Background:**

Medical teaching uses medical comics, which are combinations of images and text that depict content from everyday life in the healthcare sector. Over- or under-representation of certain people in certain roles can convey subject-specific role models and stereotypes that can influence individual training pathways. This study examines the proportion of genders and people of colour represented in comic characters, the professional roles to which they can be assigned, and the share of speech they are given.

**Method:**

The analysis included 995 German-language comics from anthologies and textbook series, with 2688 depicted characters from the period between 2002 and 2019. Criteria for categorizing roles, read gender and people of color were developed iteratively. The evaluation was carried out in a descriptive manner.

**Results:**

In the overall evaluation, the quantitatively dominant read gender was male in the roles examined (55%-88%), with especially high representation in the physician’s role (88%). Only the nursing staff were predominantly female (75%). The proportion of people of color in the roles analyzed was negligible (0-2%). The share of speech did not differ.

**Discussion:**

Medical comics used in teaching should have a balanced gender ratio, consciously depict people of color and take demographic conditions into account. This should help to create an environment in which students base their career choices on their personal skills and goals rather than on aspects such as gender or identity as people of color.

## Zusammenfassung

**Hintergrund:**

In der medizinischen Lehre werden Medizincomics eingesetzt, die als Bild- und Textkombinationen Inhalte aus dem Alltag des Gesundheitswesens darstellen. Eine Über- oder Unterrepräsentation bestimmter Personen in bestimmten Rollen kann fachspezifische Rollenbilder und Stereotype vermitteln, die individuelle Ausbildungswege beeinflussen können. Diese Studie untersucht, welchen Anteil die Geschlechter und People of Color in den Comicfiguren ausmachen, welchen fachpersonellen Rollen sie zugeordnet werden können und welchen Redeanteil sie haben.

**Methode:**

In die Analyse gingen 995 deutschsprachige Comics aus Sammelwerken und Lehrskripten ein, mit 2688 dargestellten Charakteren aus dem Zeitraum 2002-2019. Kriterien zur Kategorisierung von Rollen, gelesenem Geschlecht und People of Color wurden iterativ entwickelt. Die Auswertung erfolgte deskriptiv.

**Ergebnisse:**

In der Gesamtauswertung dominierte quantitativ in den meisten untersuchten Rollen das männlich gelesene Geschlecht (55%-88%), insbesondere in der ärztlichen Rolle (88%). Lediglich beim Pflegepersonal dominierten Figuren mit weiblich gelesenem Geschlecht (75%). Der Anteil von People of Color war in den analysierten Rollen verschwindend gering (0-2%). Die Redeanteile unterschieden sich nicht.

**Diskussion:**

In der Lehre eingesetzte Medizincomics sollten ein ausgewogenes Geschlechterverhältnis haben, People of Color bewusst darstellen und demographische Verhältnisse berücksichtigen. Das soll zur Schaffung eines Umfelds beitragen, in dem Lernende sich bei der Berufswahl an ihren persönlichen Fähigkeiten und Zielen orientieren und weniger an Aspekten wie Geschlecht oder der Identität als People of Color.

## 1. Introduction

The comic is a form of sequential art consisting of several, often drawn images with speech bubbles or descriptive text. It also includes cartoons and caricatures, which are one-panel jokes [1], [2]. Narratives depicted in this manner are typically memorable due to their on-point nature and the frequent references they make to stereotypes and role models [3].

One interface between comics and healthcare is the interdisciplinary field of “graphic medicine”, which draws upon literature, medicine, art, and clinical practice. It deals with the use of graphic narratives to depict and research medical topics and health issues. Medical comics are a core aspect of graphic medicine. They can be used to communicate complex medical issues in an understandable way, to depict aspects of illnesses or to tell therapeutically effective stories. Medical comics can thus create public awareness of health issues and support patient education, therapy and self-help [4]. Furthermore, they are used in medical training [5], [6], [7], [8], [9], [10] or can be found in self-study materials, such as those provided by Medi-Learn and Meditricks [11], [12]. In the following, the term medical comic is used for comics that depict content related to everyday healthcare. 

Previous studies on medical comics have investigated whether they support the acquisition of fine motor and communication skills, as well as empathy and the ability to reflect [13]. The use of medical comics, such as “Mom’s cancer” and “our cancer year” can facilitate students’ comprehension of the emotional and social dimensions of disease progression. The analysis of medical comics provides insights into the significance of trust and accountability in the physician-patient relationship. Furthermore, it sheds light on the potential impact of individual factors, such as gender and ethnicity, on the progression of a disease [14].

To date, there has been a lack of studies on medical comics as a medium that examines the representational diversity of the characters depicted, particularly those representing healthcare professionals. Such studies can help to expand the perception and understanding of diversity in teaching and learning materials, thus promoting the discussion of diversity.

However, there are studies that deal with cartoon characters in general. In 2004, cartoon characters from four different US daily newspapers were examined. The comics were collected over a period of one month to code the characters in terms of demographics, behaviour and activities such as sports or housework. The results of the study showed that female characters were underrepresented compared to male characters. Female characters were mostly depicted in the role of mother and wife, as well as in low-prestige occupations. Overall, the results showed that *“minorities*”, to use the terminology of the authors of the article, were barely represented in the collected comics [15].

A content analysis of American comics confirms these findings. The results show that women and people of color (PoC) comprise a significantly smaller proportion of the characters and are depicted with a lower socioeconomic status than male, white characters [16]. The term “people of color” used in the aforementioned study [16] is a self-designation of people who have experienced various forms of racism. It implies that people are not identified as part of the “white majority society” due to external characteristics and an assumed origin [17], [18]. The term “white” has evolved historically and is a social construct. The definition and understanding of “white” can vary considerably and change over time. The term is based on racialization and is used in everyday language to refer to people with external characteristics such as light skin tone, certain hair structure or facial features, which imply European descent. On the other hand, the term can also be understood in a cultural context in which norms, privileges or social status are associated with European descent [19]. 

In fact, the terms “PoC” and “white” are not ideal for coding people or comic characters based on their appearance. Appearance alone does not allow conclusions to be drawn about their ethnic origin, nor their identification with either of these terms. In a diversity analysis of comic characters, however, this is exactly what happens: Based on appearance, the characters are assigned to categories, e.g. with the labels “PoC” or “white”. These terms are also used in this article in order to use discrimination-sensitive language that takes into account that “PoC” is primarily a self-designation and “white” is a social construct.

In the aforementioned comic studies, a biased and sparse representation of PoC and female characters was observed in comparison to white male characters. An under- or non-representation of women and PoC in medical comics, whether through the appearance of the characters or their speaking parts, would not correspond to the diversity of medical personnel, thus actively excluding these groups. It could also convey discriminatory stereotypes and role models that medical students come into contact with. This is particularly relevant for women, as they are underemployed or underrepresented in many fields, including academic ones [20], [21], [22], [23], [24], [25], [26], [27]. This is also the case in medicine: although currently 64% of all medical students in Germany and almost 50% of all practicing doctors are female, women remain significantly underrepresented in certain major specialist areas, and especially in leadership positions [28], [29].

The unequal allocation of employment opportunities and training placements within companies on the basis of gender or assumed ethnic background is well documented [30], [31]. Stereotypes can also contribute to the rejection of “atypical” occupations [32]. The fear of confirming negative stereotypes (stereotype threat) can have a detrimental impact on the performance of the social group in question, as demonstrated in various scenarios to date [33], [34], [35], [36], [37], [38], [39]. Role models and stereotypes are also present among medical students, in medical teaching and in everyday clinical practice and influence the training pathway [40], [41], [42]. In particular, the work of Pelaccia et al. (2010) has shown that role models correlate with the self-efficacy expectations of medical students, which can have an impact on further training [40].

The investigation of characters in medical comics is relevant because the characters’ gender distribution, the proportion of PoC portrayed, the nature of their speaking parts and their assigned roles may convey outdated and discriminatory stereotypes and thus negatively influence the educational paths of medical students.

This leads to the formulation of the following research questions:

- What is the gender distribution of the characters in medical comics depending on the field of activity?
- What is the proportion of characters in medical comics that are recognizable as PoC, depending on the field of activity?
- What is the gender distribution among characters who are recognizable as PoC?
- What is the share of speech given to characters in medical comics dependent on gender and recognizability as PoC?

## 2. Methods

The sample was selected by purchasing printed medical comics in the form of anthologies from online mail order companies, and a textbook series for medical education. The works were identified in the period between August and October 2022. The search was carried out systematically using defined search terms (see attachment 1 ).

The year of publication of the comics, as well as the gender and birthyear of the artists, were recorded to describe the sample. Data on the artists were extracted from CVs freely available on the internet. To protect their identity, the artists were pseudonymized with consecutive identification numbers (IDs).

The operationalization of gender, recognizability as PoC and the share of the comic characters’ speech was carried out using variables and categories. Attachment 2 lists the categories for each variable. Attachment 3, tables S1 to S6 contain the criteria for the categorization. The criteria were adjusted iteratively during the data collection process. The categories “probably female” (pf) and “probably male” (pm) were introduced in order to take account of possible imprecision in the coding. The criteria for classification into pf and pm are less strict than for the gender categories “female” (f) and “male” (m). Depending on the viewpoint, pf and pm can be assigned to the categories f and m or “unknown” (u). If criteria were met for classification into both male-identified (m or pm) and female-identified (f or pf) gender categories, the figure was assigned to the “non-binary” (nb) category. The data were coded and annotated by one person.

To test the quality of the criteria, another independent person categorized a sample of 122 characters from 50 medical comics based on the criteria. The rater agreement was calculated using Cohen’s kappa and scored as <0.00=poor, 0.00-0.20=slight, 0.21-0.40=fair, 0.41-0.60=moderate, 0.61-0.80=substantial, 0.81-1.00=almost perfect [43].

“IBM SPSS Statistics version 29.0.0.0” was used to calculate descriptive statistics and to perform independence and distribution tests. If conditions for the Chi^2^ test were not met, the likelihood ratio was used. For independent samples, t-tests were performed. The significance level for the tests was set at p≤0.05.

## 3. Results

### 3.1. Descriptive statistics

2688 characters were identified and analyzed in medical comics from anthologies and a textbook series from 2015/2016. 99% of the comics were drawn by men. The characters were mostly assigned to the categories m and pm. 46% of the identified roles were assigned to “physician staff”, “nursing staff” and “other healthcare professionals”. Further information on the sample can be found in table 1 (Tab. 1).

The coding of a sub-sample by two independent persons resulted in substantial rater agreement for all variables (see table 2 (Tab. 2)).

Table 3 (Tab. 3) lists the artists’ IDs and the relative proportions of comic characters per artist. 

In the comics from the anthologies (n=2322 characters), 42% of the characters were created by artist 4 and 12% by artist 27. A test of independence shows that the comics of artist 4 differ from the comics of other artists in terms of the distribution of the characters across the categories of the variables. A second test of independence was carried out excluding the works of artist 4, which shows that the comics by artist 27 also differ from the works of the other artists. The comics of artists 4 and 27 were therefore analyzed separately from the comics of the other artists. The separate analysis of the medical comics shows that the works of artists 4 and 27 differ from the comics of all the other artists, but that the interpretation of the results comes down to the same aspects despite the differences. For this reason, the results relating to the collective works are considered together.

### 3.2. Gender ratio in medical comics depending on areas of activity and roles

**Figure 1:**
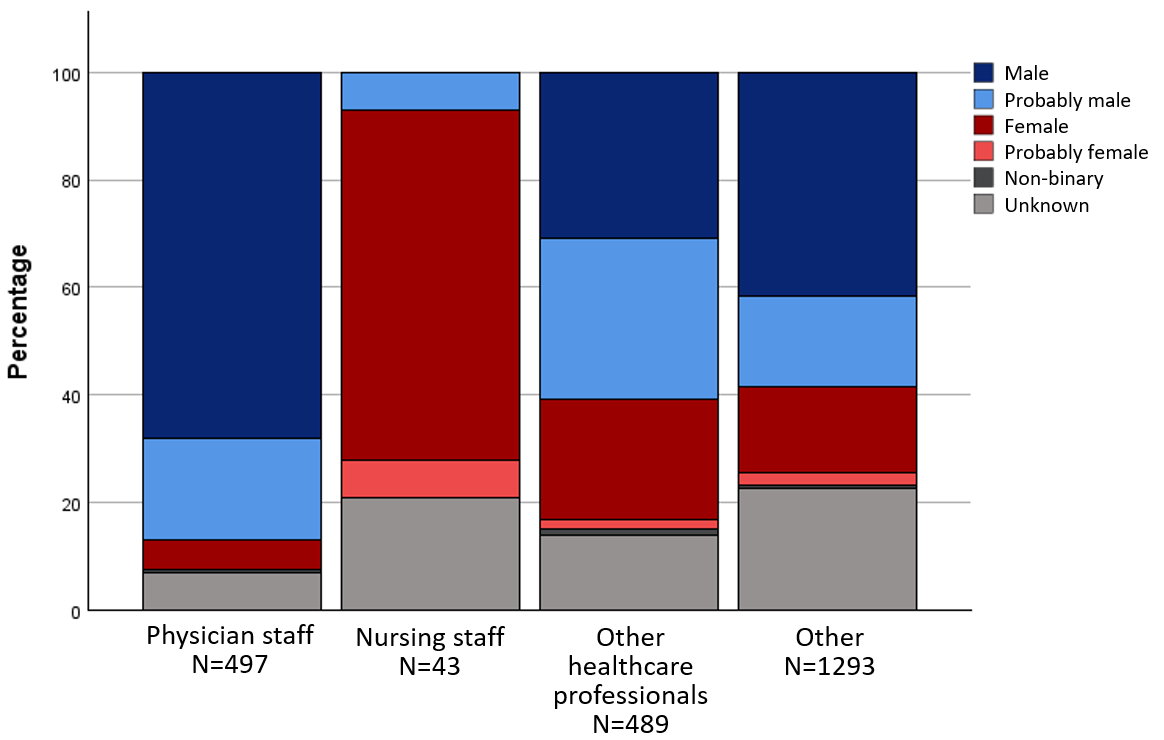
Gender distribution in the basic roles in the comics from anthologies

**Figure 2:**
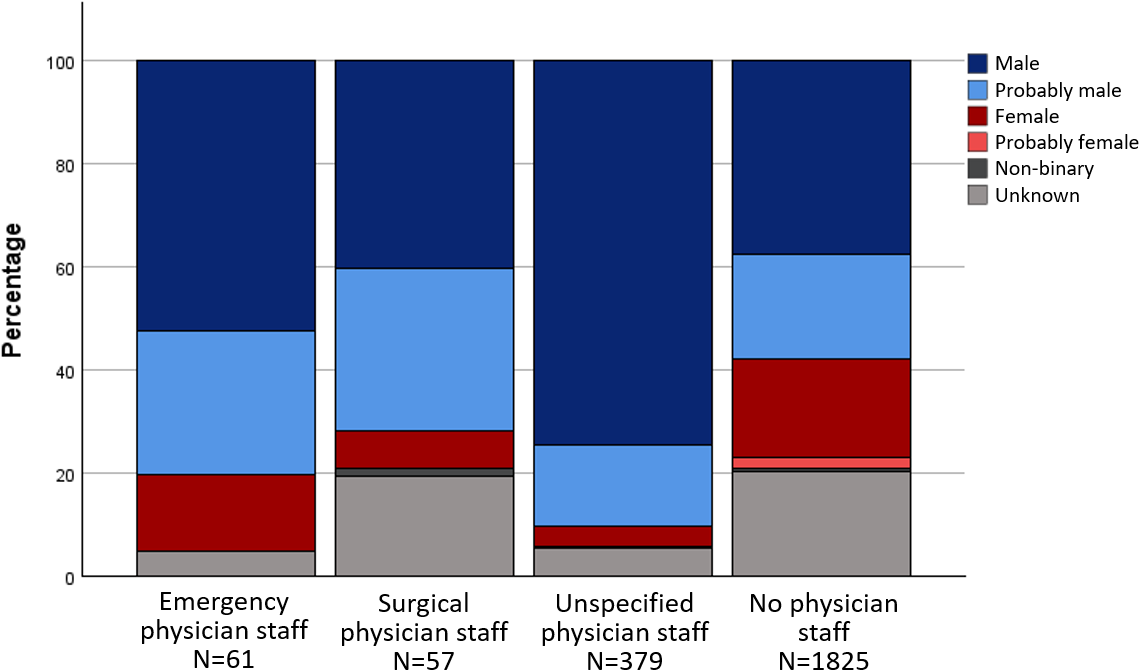
Gender distribution in the specialist physician roles in the comics from anthologies

**Figure 3:**
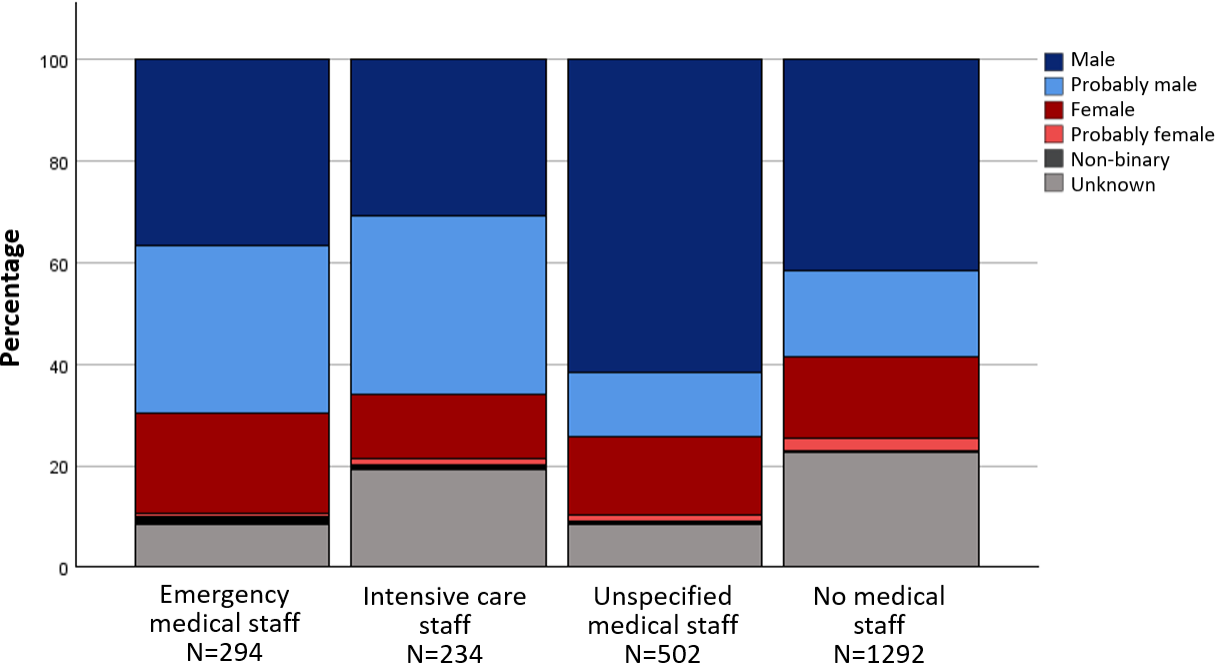
Gender distribution of the roles of qualified personnel in the comics from anthologies

In the anthologies, male-identified characters were predominantly assigned to the basic roles of “physician staff”, “other healthcare professionals” and the categories of “specialist physician role” and “qualified personnel”. Characters in the role of "nursing staff", on the other hand, were mainly assigned to the gender categories f and pf. The gender distribution across different areas of activity and roles is shown in figure 1 (Fig. 1), figure 2 (Fig. 2) and figure 3 (Fig. 3) for the anthologies.

**Figure 4:**
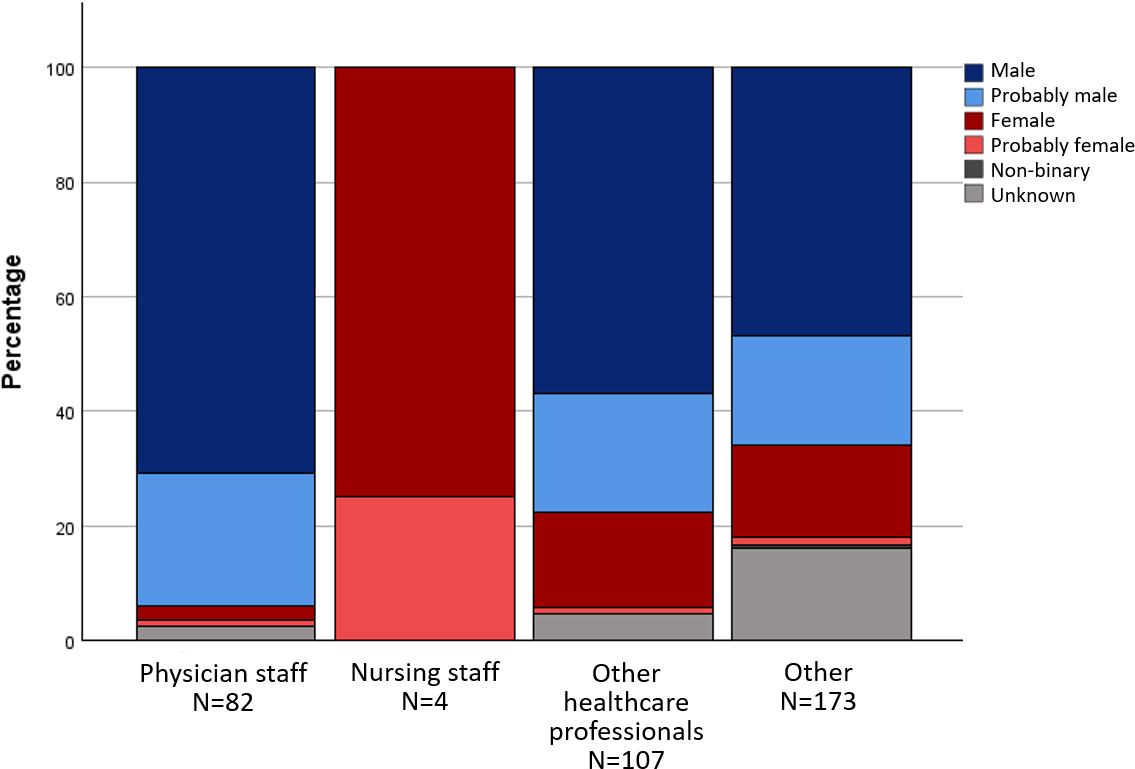
Gender distribution in the basic roles in the comics from the textbook series

**Figure 5:**
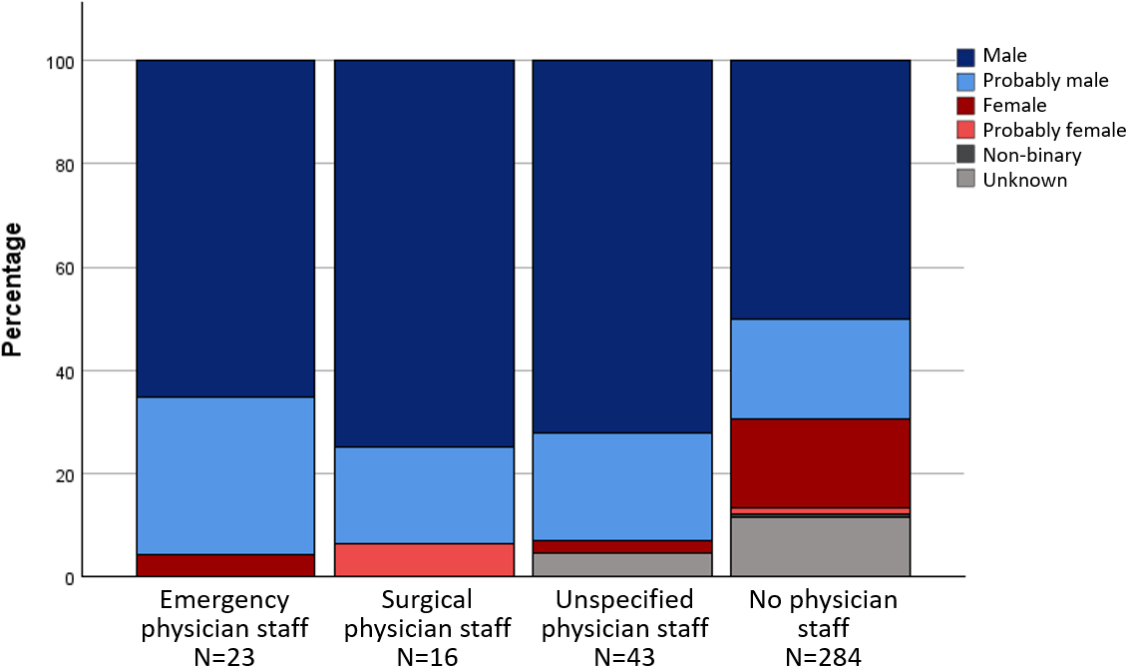
Gender distribution in the specialist physician roles in the comics from the textbook series

**Figure 6:**
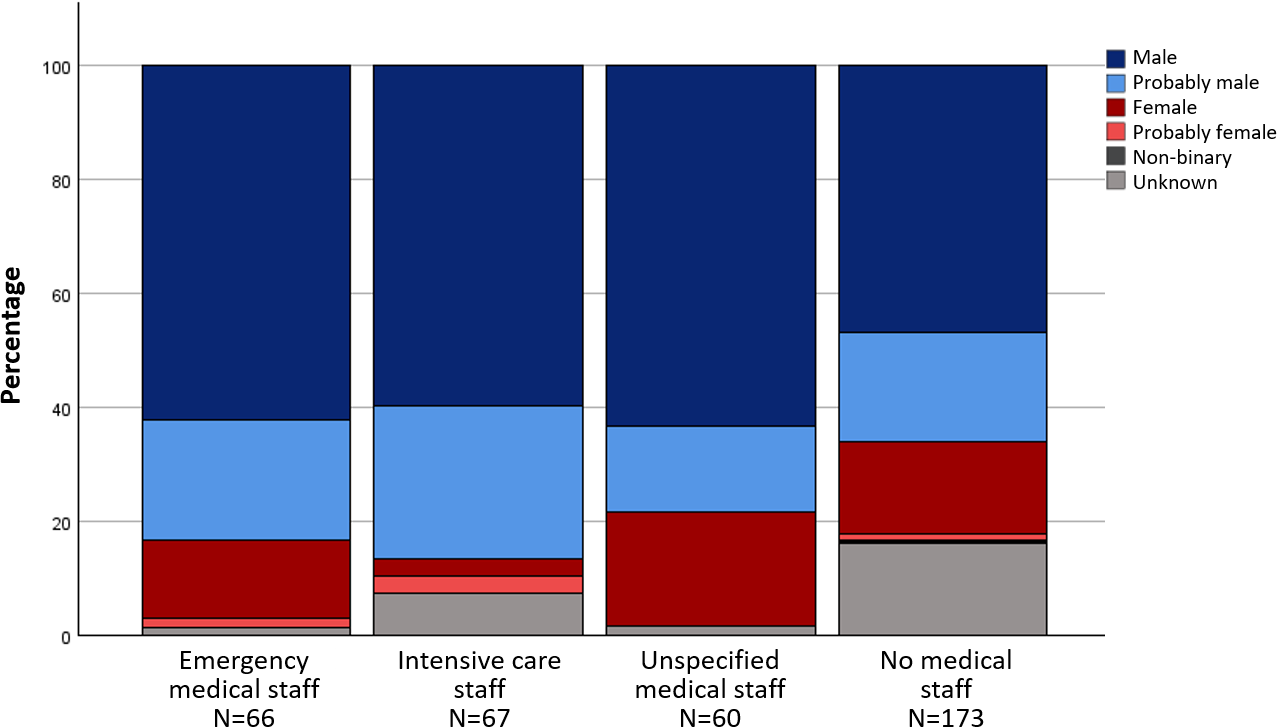
Gender distribution of the roles of qualified personnel in the comics from the textbook series

The characters in the textbook series were mostly assigned to the categories m and pm in the basic roles. One exception is the role of “nursing staff”. Four characters were assigned to “nursing staff” and at the same time to the categories f and pf. Figure 4 (Fig. 4), figure 5 (Fig. 5) and figure 6 (Fig. 6) illustrate the gender proportions depending on the areas of activity and roles in the textbook series.

### 3.3. Proportion of figures recognizable as PoC depending on areas of activity and roles

Characters recognizable as PoC made up 1% in both the comics from the anthologies (n=26) and in the textbook series (n=4) (see table 4 (Tab. 4) and table 5 (Tab. 5)). For this reason, a more in-depth analysis depending on the different roles was not carried out for either data pool. The proportion of PoC characters in the works of artist 27 and the other artists was also 1%. Only in the comics of artist 4 was their proportion 2%.

### 3.4. Gender distribution among figures recognizable as PoC

In the anthologies, characters assigned to the category “recognizable as PoC” (n=26) were predominantly read as male. The ratio between PoC characters read as male and female did not differ from that of characters who were not recognizable as PoC, excluding the gender categories “unknown” (17%, n=404) and “non-binary” (1%, n=15) (see table 6 (Tab. 6)). 

In the textbook series, a total of four characters were assigned to the category “recognizable as PoC” (n=4). Two of these were coded f and two m (see table 6 (Tab. 6)). The ratio between PoC characters read as male and female thus differs from that of characters not recognizable as PoC, not considering the gender categories “unknown” (10%, n=35) and “non-binary” (0.3%, n=1).

### 3.5. Share of speech of the characters based on the variables gender and PoC 

When analyzing the comics from the anthologies, there were no differences in the share of speech depending on the variables gender and PoC (see table 7 (Tab. 7)).

In the comics of the textbook series, the share of speech does not differ depending on gender (see table 8 (Tab. 8)). Due to the small number of identified characters (n=4), the share of PoC characters’ speech was not analyzed in the textbook series.

Separate analysis of the works of artists 4 and 27, which make up the largest proportion of the data pool, reveals subtle differences in the gender distribution. Analyzing the share of speech in the comics by cartoonist 4 showed that male characters speak more frequently and have a greater share of speech than female characters, measured by the number of words in integer numbers. The analysis of the share of speech in the comics by cartoonist 27 showed that female characters have a higher share of speech than male characters, measured by the number of words in integer numbers. There is no difference in the share of speech per contribution depending on gender.

## 4. Discussion

The analysis of the medical comics shows that the characters in the basic and specialist physician role, as well as healthcare professionals, are predominantly depicted as male. The gender categories “female” and “probably female” dominate among the characters in the role of “nursing staff”. Only 1% of the characters in the anthologies and textbook series can be categorized as PoC. There was no significant difference in the share of speech depending on gender and recognizability as PoC. In summary, the medical comics analyzed most frequently depict white male characters. The nursing staff are drawn as white and female. PoC, particularly women, are underrepresented in all areas.

Strengths and limitations of the study are discussed in order to classify the results. The search for medical comics was carried out systematically and carefully, resulting in a large pool of data. A representative sample of German-language medical comics was to be identified through defined search terms and the use of various online mail order companies. Nevertheless, it is possible that not all medical comics eligible for analysis were included.

The variables, categories and categorization criteria were developed iteratively by two people in exchange for a person-independent assignment of the characters. The interindividual independence is demonstrated by the high degree of rater agreement of a sample of the comic characters by two independent persons, which makes a study limitation due to inadequate definition of the categories and incorrect assignment of the characters unlikely.

The results of our analysis are in line with the findings of American comic studies, which found similar gender ratios and proportions of PoC in comic characters [15], [16]. 

However, it is not only the proportion of female characters and those who were recognizable as PoC that is decisive, but also the area of activity in which they are portrayed. Characters who were portrayed in the basic role of “physician staff” were mostly “male” and “probably male”. In 2022, 49% of working doctors in Germany were women [44]. The characters identified as surgeons were also mostly depicted as “male” or “probably male”. Although there are more men than women working in surgery in Germany, more and more women are choosing to become surgeons. This is evidenced by the increasing number of working female surgeons in the German national physician statistics [29], [44]. Characters assigned to the emergency medical staff category were also mostly “male” and “probably male”. The actual gender ratio of emergency physicians is more difficult to answer, as the German national physician statistics do not break down the list of auxiliary advanced education in emergency medicine by gender. In a 2013 survey on the demographics, training and professional experience of emergency doctors in Germany, 80% of the 1991 study participants were male [45]. It can be assumed that the proportion of women among emergency physicians has increased since then.

The characters read as “nursing staff” were predominantly depicted as “female” and “probably female”. The statistics of the German Federal Employment Agency show similar ratios for nursing care in 2023 [46]. Around 4 out of 5 nursing specialists are female, but the number of male workers has also increased at an above-average rate over the years.

Only a small proportion of all characters analyzed were coded with the category “recognizable as PoC”. A comparison with the actual situation among healthcare professionals is difficult, as no statistics reflect the number of people who identify as PoC. Although migrant background and foreign nationality are statistically recorded for physician and nursing staff, these figures do not allow conclusions to be drawn about identification as a PoC. However, based on our personal experience in the healthcare system, it can be said that the demographics of German society and the physician and nursing staff are more diverse than portrayed in the comics examined.

The analysis of the medical comics and the comparison with current figures on healthcare personnel make it clear that gender relations are portrayed too one-sidedly and that the proportion of PoC figures probably does not reflect current developments. In particular, medical comics that are developed for educational purposes should address demographic developments in physician and nursing staff. When designing comics, attention should also be paid to portraying a variety of characters in different roles. A balanced gender distribution is just as important as the deliberate portrayal of PoC. The entire range of medical specialties and tasks should also be addressed and visualized.

Representational diversity should help create an environment that enables learners to find the career path that suits their personal abilities and goals, and is less dependent on aspects such as gender or identity as a PoC. However, existing medical comics are also valuable material. They can be used to actively work on the aspects identified in the analysis with the students, to reflect on stereotypes and role models and to address contradictions and discrimination. The analysis of medical comics as part of teaching and learning materials is an important component in the further development of medical education and contributes to reflecting on structures and uncovering both facilitating and inhibiting factors. Future studies could investigate which stereotypes and role models exist among teachers and medical students, and which linguistic and visual means are used to address these stereotypes in medical comics and other teaching and learning materials.

## Authors’ ORCIDs

- Cyrus Amin Parsa: [0009-0004-7280-8348]
- Johanna Hirsch: [0009-0002-9818-5287]
- Katrin Schüttpelz-Brauns: [0000-0001-9004-0724]

## Competing interests

The authors declare that they have no competing interests. 

## Supplementary Material

Online mail order companies and search terms used for the sample selection

Variables and categories for classifying individual characters

Additional tables

## Figures and Tables

**Table 1:**
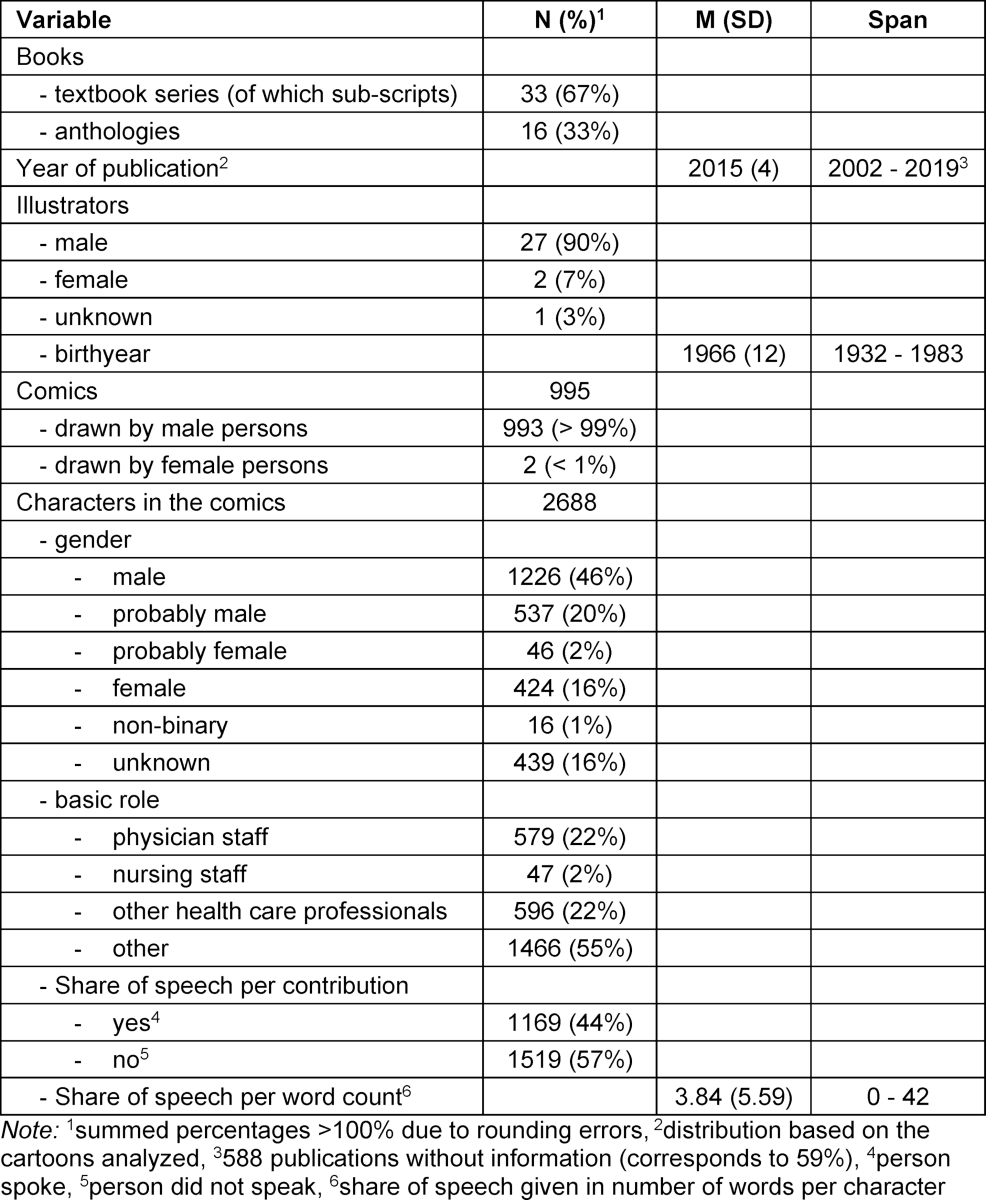
Sample description

**Table 2:**
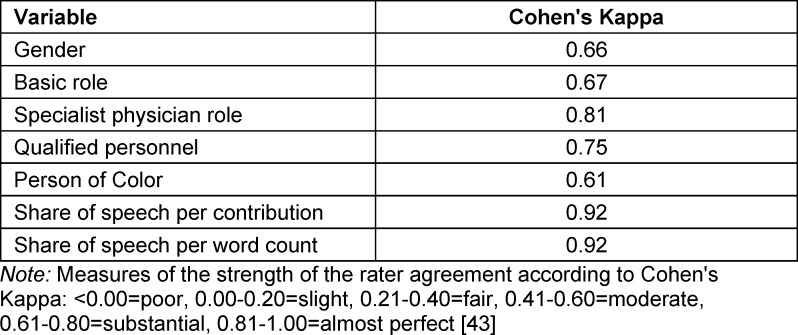
Rater agreement according to Cohen’s Kappa

**Table 3:**
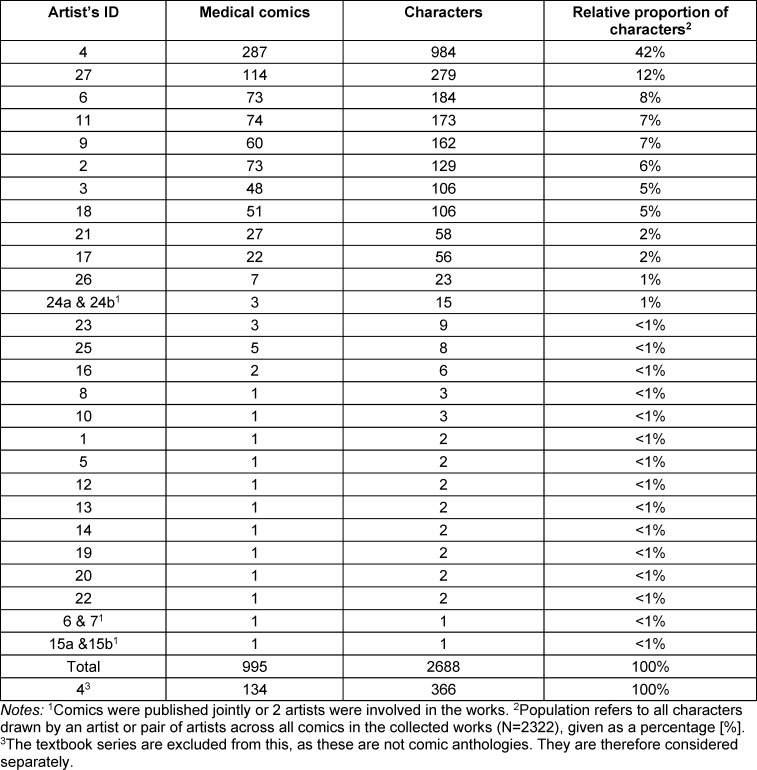
Number of medical comics and characters per artist

**Table 4:**
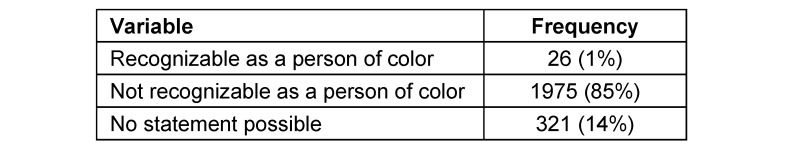
Proportion of characters identified as people of color. Medical comics from the anthologies

**Table 5:**
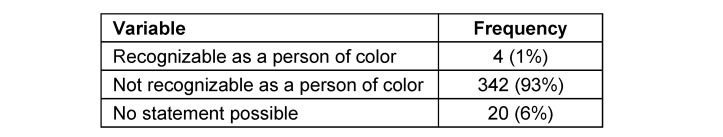
Proportion of characters identified as people of color. Medical comics in the textbook series

**Table 6:**
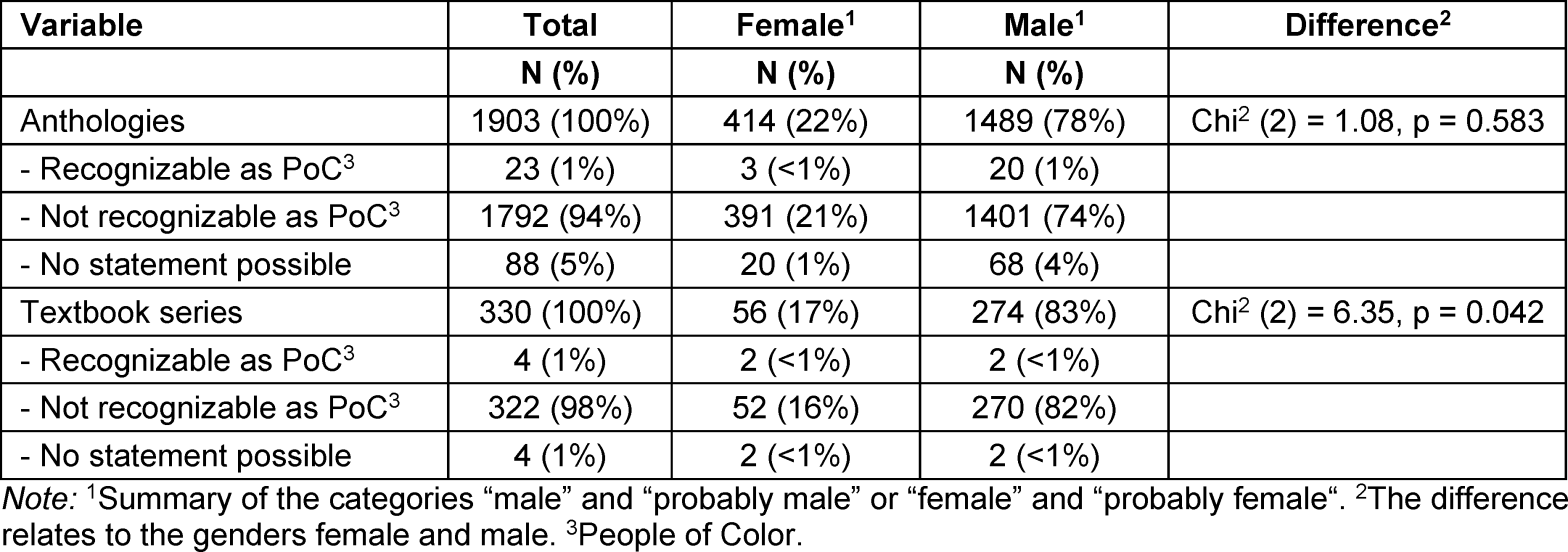
Gender distribution in anthologies and in the textbook series among characters who were identifiable as people of color

**Table 7:**
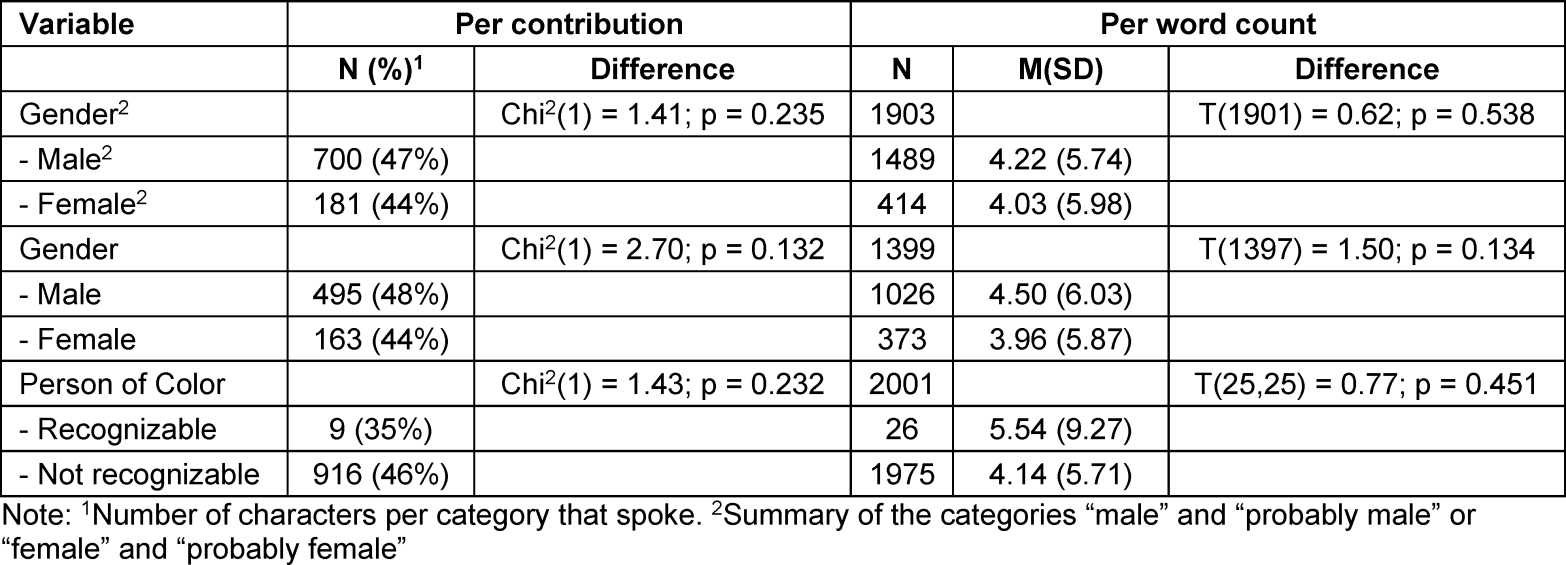
Proportion of share of speech per contribution and per word count depending on gender and recognizability as a people of color in comics from the anthologies

**Table 8:**
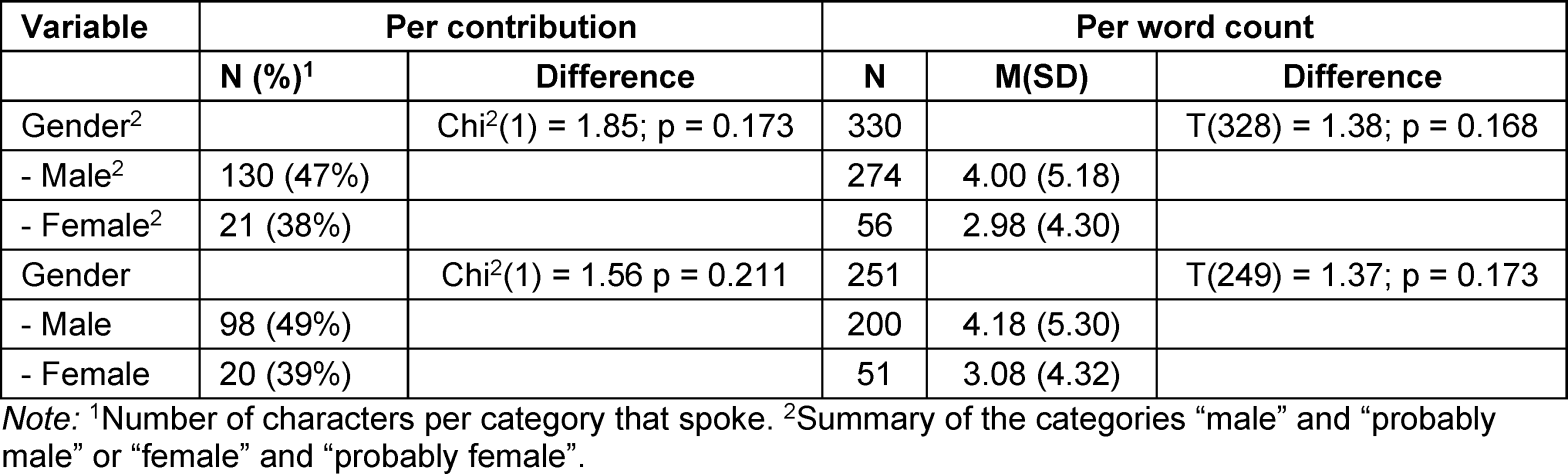
Proportion of share of speech per contribution and per word count depending on gender in comics from the textbook series

## References

[1] Packard S, Stephan D, Katerina K, Daniel S (2009). Was ist ein Cartoon? Psychosemiotische Überlegungen im Anschluss an Scott McCloud.

[2] Zimmermann & Heitmann GmbH (2024). Comic.

[3] Giardinelli A (2016). Die Dekonstruktion von Stereotypen in Comics anhand des Beispiels „Sturmtruppen“ von Bonvi.

[4] Krüger-Fürhoff IM (2020). Illness narratives in comics: using graphic medicine in the medical humanities. Wien Klin Wochenschr.

[5] Green MJ, Myers KR (2010). Graphic medicine: use of comics in medical education and patient care. BMJ.

[6] Lesińska-Sawicka M (2023). Using graphic medicine in teaching multicultural nursing: a quasi-experimental study. BMC Med Educ.

[7] Babaian CS, Chalian AA (2014). "The thyroidectomy story": comic books, graphic novels, and the novel approach to teaching head and neck surgery through the genre of the comic book. J Surg Educ.

[8] Green MJ (2013). Teaching with comics: a course for fourth-year medical students. J Med Humanit.

[9] Shin D, Kim DH, Park JS, Jang HG, Chung MS (2013). Evaluation of anatomy comic strips for further production and applications. Anat Cell Bioly.

[10] Masel EK, Praschinger A (2020). Using comics to teach medical humanities. Wien Klin Wochenschr.

[11] MEDI-LEARN (2024). MEDI-LEARN Kurse.

[12] Meditricks (2024). Medizin meistern.

[13] Consorti F, Fiorucci S, Martucci G, Lai S (2023). Graphic Novels and Comics in Undergraduate and Graduate Medical Students Education: A Scoping Review. Eur J Investig Health Psychol Educ.

[14] Squier SM (2007). Beyond Nescience: the intersectional insights of health humanities. Perspect Biol Med.

[15] Glascock J, Preston-Schreck C (2004). Gender and Racial Stereotypes in Daily Newspaper Comics: A Time-Honored Tradition?. Sex Roles.

[16] Facciani M, Warren P, Vendemia J (2015). A Content-Analysis of Race, Gender, and Class in American Comic Books. Race Gender Class.

[17] Bundeszentrale für politische Bildung (2018). LSBTIQ-Lexikon: of Color, People / Queers (PoC, QPoC).

[18] Ha KN (2013). 'People of Color' als Diversity-Ansatz in der antirassistischen Selbstbenennungs- und Identitätspolitik.

[19] Gallagher CA, Batur P, Feagin JR (2018). White.

[20] Rudman LA, Phelan JE (2010). The effect of priming gender roles on women’s implicit gender beliefs and career aspirations. Soc Psychol.

[21] Quimby JL, DeSantis AM (2006). The Influence of Role Models on Women's Career Choices. Career Dev Q.

[22] Steffens MC, Ebert ID (2016). Auswirkungen von Vorbildern. Frauen – Männer – Karrieren Eine sozialpsychologische Perspektive auf Frauen in männlich geprägten Arbeitskontexten.

[23] Burrows D, Pietri E, Johnson I, Ashburn-Nardo L (2022). Promoting Inclusive Environments: In-group Organizational Endorsement as a Tool to Increase Feelings of Identity-Safety among Black Women. Sex Roles.

[24] Cook A, Glass C (2014). Women and Top Leadership Positions: Towards an Institutional Analysis. Gender Work Organ.

[25] Acker J (2006). Inequality Regimes: Gender, Class, and Race in Organizations. Gender Soc.

[26] Turner CS, González JC, Wood JL (2008). Faculty of color in academe: What 20 years of literature tells us. J Div High Educ.

[27] Kracke N (2016). Unterwertige Beschäftigung von AkademikerInnen in Deutschland. Die Einflussfaktoren Geschlecht, Migrationsstatus und Bildungsherkunft und deren Wechselwirkungen. Soz Welt.

[28] Statistisches Bundesamt (2023). Anzahl der Studierenden im Fach Humanmedizin in Deutschland nach Geschlecht in den Wintersemestern von 2010/2011 bis 2022/2023.

[29] Bundesärztekammer (2023). Ärztestatistik zum 31. Dezember 2023.

[30] Imdorf C, Scherr A, El-Mafaalani A, Yüksel G (2017). Diskriminierung in der beruflichen Bildung.

[31] Beicht U, Granato M (2010). Ausbildungsplatzsuche: Geringere Chancen für junge Frauen und Männer mit Migrationshintergrund.

[32] Busch A (2013). Die berufliche Geschlechtersegregation in Deutschland; Ursachen, Reproduktion, Folgen.

[33] Aronson E, Wilson TD, Akert RM, Fischer P, Jander K, Krueger JI (2008). Bedrohung durch Stereotype.

[34] Aronson JM, Lustina MJ, Good C, Keough K, Steele CM, Brown J (1999). When white men can’t do math: Necessary and sufﬁcient factors in stereotype threat. J Exp Soc Psychol.

[35] Aronson JM, Quinn D, Spencer S, Swim JK, Stangor C (1998). Stereotype threat and the academic underperformance of women and minorities.

[36] Steele CM, Aronson JM (1995). Stereotype threat and the intellectual test performance of African-Americans. J Pers Soc Psychol.

[37] Steele CM, Aronson JM, Aronson E (1994). Stereotype vulnerability and intellectual performance.

[38] Spencer SJ, Steele CM, Quinn DM (1999). Stereotype Threat and Women's Math Performance. J Exp Soc Psychol.

[39] Stone J, Lynch CI, Sjomeling M, Darley JM (1999). Stereotype threat effects on Black and White athletic performance. J Pers Soc Psychol.

[40] Pelaccia T, Delplanq H, Triby E, Bartier JC, Leman C, Hadef H, Pottecher T, Dupeyron JP (2010). Gender stereotypes: an explanation to the underrepresentation of women in emergency medicine. Acad Emerg Med.

[41] Samuriwo R, Patel Y, Webb K, Bullock A (2020). ‘Man up’: Medical students’ perceptions of gender and learning in clinical practice: A qualitative study. Med Educ.

[42] Woolf K, Cave J, Greenhalgh T, Dacre J (2008). Ethnic stereotypes and the underachievement of UK medical students from ethnic minorities: qualitative study. BMJ.

[43] Landis JR, Koch GG (1977). The measurement of observer agreement for categorical data. Biometrics.

[44] Bundesärztekammer (2022). Ärztestatistik zum 31. Dezember 2022.

[45] Ilper H, Kunz T, Walcher F, Zacharowski K, Byhahn C (2013). Demografie, Ausbildung und Erfahrung der Notärzte in Deutschland: www.notarztfragebogen.de. Dtsch Med Wochenschr.

[46] Bundesagentur für Arbeit (2023). Arbeitsmarktsituation im Pflegebereich.

