# Supplementary material for: Representation of gender and people of color among healthcare professionals in medical comics – a document analysis
Source: GMS J Med Educ. 2025 Feb 17;42(1):Doc2. doi: 10.3205/zma001726 (PMC12086249; doi:10.3205/zma001726)
Supplement: Online mail order companies and search terms used for the sample selection [file JME-42-2-s-001.pdf]

Attachment 1: Online mail order companies and search terms used for the sample selection

Online retailer	Search terms
www.amazon.de<br>www.medimops.de<br>www.weltbild.de/buecher<br>www.buecher.de<br>www.deutsche-buchhandlung.de<br>www.thalia.de<br>www.ebay.de<br>www.ebay-kleinanzeigen.de	Doctor cartoons<br>Doctor comics<br>Doctors cartoons<br>Doctors comics<br>Female doctor cartoons<br>Female doctor comics<br>Female doctors cartoons<br>Female doctors comics<br>Medicine cartoons<br>Medicine comics<br>Nursing cartoons<br>Nursing comics

Note: The search period was August to October 2022. The search was conducted using search terms in the German language.

Attachment 1 to Amin Parsa C, Hirsch J, Schüttpelz-Brauns K. *Representation of gender and people of color among healthcare professionals in medical comics – a document analysis*. GMS J Med Educ. 2025;42(1):Doc2. DOI: 10.3205/zma001726
