# Supplementary material for: Representation of gender and people of color among healthcare professionals in medical comics – a document analysis
Source: GMS J Med Educ. 2025 Feb 17;42(1):Doc2. doi: 10.3205/zma001726 (PMC12086249; doi:10.3205/zma001726)
Supplement: Variables and categories for classifying individual characters [file JME-42-2-s-002.pdf]

Attachment 2: Variables and categories for classifying individual characters

Variable	Category
Gender	Female<br>Probably female<br>Probably male<br>Male<br>Non-binary<br>Unknown
Basic role	Physician staff<br>Nursing staff<br>Other healthcare professionals<br>Other
Specialist physician role	Surgical physician staff<br>Emergency physician staff<br>Unspecified physician staff<br>No physician staff
Qualified personnel	Emergency medical staff<br>Intensive care staff<br>Unspecified medical staff<br>No medical staff
Person of color	Recognizable as a person of color<br>Not recognizable as a person of color<br>No statement possible
Share of speech per word count	Numerical[1]
Share of speech per contribution	Yes[2]<br>No[3]

Note. [1] Measured by the number of words in integer numbers. [2]Character spoke. [3]Character did not speak.
