# Supplementary material for: Representation of gender and people of color among healthcare professionals in medical comics – a document analysis
Source: GMS J Med Educ. 2025 Feb 17;42(1):Doc2. doi: 10.3205/zma001726 (PMC12086249; doi:10.3205/zma001726)
Supplement: Additional tables [file JME-42-2-s-003.pdf]

Attachment 3: Additional tables

Table S1: Criteria for categorizing the gender variable

Category	Female (f)	Male (m)	Probably female (pf)	Probably male (pm)	Non-binary (nb)	Unknown (u)
Criteria	(Personal) addressing/own or third-party designation through comic text or commentary by the illustrator<br>Representation of primary female sexual characteristics<br>Female breasts<br>Pregnancy<br>Three (3) fulfilled criteria from the following are considered sufficient here:<br>- Long hair/hairnet[1]<br>- Drawn eyelashes<br>- Earrings/ornamental necklace<br>- Lipstick/make-up (clown make-up does not count)<br>- Hair circlet/ hair tie<br>- Being addressed as part of a group (e.g. "Hey girls")<br>- Dress/skirt/women's folk costume<br>- Stockings<br>- Women's boots/shoes with high heels<br>- Carrying a women's handbag<br>- Women's hat	(Personal) addressing/own or third-party designation through comic text or commentary by the illustrator<br>Representation of primary male sexual characteristics<br>Beard/beard stubble/chops<br>Bald/balding/receding hairline (unless it is clear that the person has recently had surgery on the skull, e.g. sutures on the head, etc. or oncological therapy)<br>Chest hair<br>Three (3) fulfilled criteria from the following are considered sufficient here:<br>- Short hair/cap[1]<br>- Eyes without eyelashes<br>- Flat chest<br>- Being addressed as part of a group (e.g. "Hey guys")<br>- Tie / bow tie<br>- Hairy limbs<br>- Men's hat<br>- Suit/Men's folk costume	A categorization in f was not possible based on the criteria<br>PLUS<br>Two (2) fulfilled criteria from the following are considered sufficient here:<br>- Long hair<br>- Drawn eyelashes<br>- Earrings/ornamental necklace<br>- Lipstick<br>- Hair circlet/ hair tie<br>- Being addressed as part of a group (e.g., „Hey girls“)<br>- Stockings<br>- Women's boots/shoes with high heels<br>- Dress/skirt/ women's folk costume<br>- Carrying a women's handbag<br>- Women's hat	Categorization in m was not possible based on the criteria<br>PLUS<br>Two (2) fulfilled criteria from the following are considered sufficient here:<br>- Short hair<br>- Eyes without eyelashes<br>- Flat chest<br>- Being addressed as part of a group (e.g. "Hey guys")<br>- Tie / bow tie<br>- Hairy limbs<br>- Men's hat<br>- Suit/ Men's folk costume	(Personal) addressing/own or third-party designation through comic text or commentary by the illustrator<br>Criteria were met for 2 categories of m, f, pm and pf	(Personal) addressing/own or third-party designation through comic text or commentary by the illustrator<br>Categorization into m, f, pm, pf, and nb was not possible based on the criteria

Notes: [1] Hair under a hairnet is counted as long, under a cap as short.

Table S2: Criteria for categorizing the basic role variable

**Physician staff (Ps)**[1]	**Nursing staff (Ns)**[1]	**Other healthcare professionals (Ohp)**[1,2]	**Other (O)**[1]
(Personal) addressing/own or third-party designation through comic text or commentary by the illustrator<br><br>Name tags with "Dr.", "NA[3] ", "Emergency doctor" or "Emergency physician"<br><br>Categorization as "Sps[3] " or "Eps[3] "<br><br>Addressing/designation with specialist title<br><br>Stethoscope<br><br>Forehead mirror<br><br>Execution of strictly medical activities reserved for physicians:<br>- Anamnesis<br>- Making an indication<br>- Patient examination including invasive tests<br>- Establishing a diagnosis<br>- Patient education and counseling<br>- Decide on treatment plan<br>- Implementation of invasive therapies including the core service of surgical interventions<br>- Doctor´s visit<br><br>White/blue/green medical uniform[4]<br>PLUS all of the following criteria is considered sufficient:<br>- Surgical cap/hairnet<br>- Face mask<br>- Blood-stained gloves<br>- Close proximity to operating room/operating table or intensive care unit	(Personal) addressing/own or third-party designation through comic text or commentary by the illustrator<br><br>Name tags with "nursing specialist", "caregiver" or "(male) nurse"<br><br>Wearing a nurse's cap<br><br>ATTENTION!<br>If designated as a nurse in close proximity of the operating room/operating table, then classification as Ohp	(Personal) addressing/own or third-party designation through comic text or commentary by the illustrator<br><br>Medical cross symbol<br><br>Designation with "emergency service", "RD[3]" or similar<br><br>Designation as Laboratory staff<br><br>Wearing medical equipment<br><br>Operating a medical device<br><br>Driving an (emergency) ambulance, emergency motorcycle, (emergency) rescue helicopter or rescue transport vehicle or to be a co-pilot/co-driver of these vehicles<br><br>Visibility clothing in orange/flame red with reflective stripes<br><br>Designation as surgical assistant, surgical technologist, operating room technician, operating room assistant or OR[3] nurse<br><br>Designation as a nurse in close proximity of the operating room/operating table<br><br>Students in clinical internship / the practical year, clinical trainee, Students in clinical rotations<br><br>Serving patients/accompanying persons at the registration desk<br><br>Assisting the Ps or Ns<br><br>Performing psychotherapy<br><br>White/blue/green medical uniform[4]<br><br>PLUS one of the following criteria is considered sufficient:<br>- Provider of medical service(s)<br>- Surgical cap/hairnet<br>- Face mask<br>- Close proximity to operating room/operating table, (intensive care) ward, ambulance[3], practice or waiting room<br><br>Working clothes PLUS one of the following criteria is sufficient:<br>- Call someone in the waiting room in an outpatient setting	(Personal) addressing/own or third-party designation through comic text or commentary by the illustrator<br><br>Categorization into Ps, Ns and Ohp was not possible based on the criteria

Notes: [1] If several categories apply, then allocation to the category with higher priority, unless the context does not allow it. Highest priority: Ps. then Ns, then Ohp, then lowest priority: O.[2] to Ohp: Opticians and pharmacists do not fall under Ohp. [3] Abbreviations in alphabetical order: Eps = Emergency physician staff, NA = German abbr. for "Notarzt", which translates to emergency doctor, OR = Operating Room, RD = German abbr. for "Rettungsdienst", which translates to emergency service, Sps = surgical physician staff, [4] For white/blue/green uniforms: 1: A disposable protective gown (e.g.: isolation - gowns for visitors, over clothing of a different color other than blue/green) is not considered work clothing. 2: If the comic is black-and-white, medical uniforms are sufficient regardless of color and shade.

Table S3: Criteria for categorizing the specialist physician role variable

**Category**	**Surgical physician staff (Sps)**	**Emergency physician staff (Eps)**	**Unspecified physician staff (Ups)**	**No physician staff (Nps)**
Criteria	(Personal) addressing/(own or other) designation through comic text or commentary by the illustrator<br>Performing surgical procedures in white/blue/green medical uniforms[1]<br>White/blue/green medical uniform[1] PLUS all of the following criteria are considered sufficient:<br>- Surgical cap/hairnet<br>- Face mask<br>- Bloody gloves<br>- Close proximity to operating room/operating table or intensive care unit	(Personal) addressing/(own or other) designation through comic text or commentary by the illustrator<br>Name tags with "NA[2]", "emergency doctor"<br>Already categorized as Ps[2] PLUS one of the following criteria is considered sufficient:<br>- Wearing emergency service uniform or visibility clothing in orange/flame red with reflective stripes	(Personal) addressing/(own or other) designation through comic text or commentary by the illustrator<br>Already categorized as Ps[2] without possible assignment to Sps or Eps	(Personal) addressing/(own or other) designation through comic text or commentary by the illustrator<br>Not categorized as Ps[2]

Notes: [1]For white/blue/green uniforms: A disposable protective gown (e.g.: isolation-gowns for visitors, over clothing of a different color other than blue/green) is not considered work clothing. If the comic is black-and-white, medical uniforms are sufficient regardless of color and shade., [2] Abbreviations in alphabetical order: NA = German abbr. for “Notarzt”, which translates to emergency doctor, Ps = physician staff.

Table S4: Criteria for categorizing the qualified personnel variable

Category	Emergency medical staff (Ems)[1]	Intensive care staff (Ics)[1,2]	Unspecified medical staff (Ums)[1]	No medical staff (Nms)
Criteria	(Personal) addressing/(own or other) designation through comic text or commentary by the illustrator<br><br>Already categorized as Eps[3]<br><br>Driving an (emergency) ambulance, emergency motorcycle, (emergency) rescue helicopter or rescue transport vehicle or to be a co-pilot/co-driver of these vehicles<br><br>Wearing emergency service uniform or visibility clothing in orange/flame red with reflective stripes<br><br>Provide medical services or operate/carry medical equipment in the ambulance<br><br>White/blue/green[4] medical uniforms in close proximity of the ambulance<br><br>Designated as "Emergency service", "RD[3]" or similar<br><br>First-aiders in medical uniform<br><br>Transporting patients on a rescue stretcher	(Personal) addressing/(own or other) designation through comic text or commentary by the illustrator<br><br>Designation as an anesthesiologist / surgical assistant, surgical assistant, surgical technologist, operating room technician, operating room assistant or OR[3] nurse<br><br>Designation as a nurse in close proximity of the operating room/operating table<br><br>Already categorized as Sps[3]<br><br>White/blue/green medical uniform[4] PLUS one of the following criteria is considered sufficient:<br>- Surgical cap/hairnet<br>- Face mask (except for during dental procedures)<br>- Close proximity to operating room/operating table or intensive care unit	(Personal) addressing/(own or other) designation through comic text or commentary by the illustrator<br><br>Provider of medical service(s)<br>Operating medical equipment<br>Carrying medical equipment<br>Wearing the medical cross symbol<br>Already categorized as Ps[3], Ns[3], Ohp[3]<br>Students in the practical year, clinical trainee<br>Laboratory staff	(Personal) addressing/(own or other) designation through comic text or commentary by the illustrator<br><br>Categorization into Ems, Ics and Ums was not possible based on the criteria

Notes: [1]If more than one category applies, then assignment to the category with higher priority, unless the context does not allow it. Highest priority: RP. Medium priority: Ics. Low priority: uMP. [2]Laboratory staff does not fall under Ics. [3]Abbreviations in alphabetical order: Eps = Emergency physician staff, OR = Operating Room, RD = German abbr. for “Rettungsdienst”, which translates to emergency service, Sps = surgical physician staff, Ps = physician staff, Ns = nursing staff, Ohp = Other healthcare professionals, [4] For white/blue/green uniforms: A disposable protective gown (e.g.: isolations gowns for visitors, over clothing of a different color other than blue/green) is not considered work clothing. If the comic is black-and-white, medical uniforms are sufficient regardless of color and shade.

Table S5: Criteria for categorizing the Person of Color variable

Recognizable as a person of color	Not recognizable as a person of color	No statement possible
(Personal) addressing/ (own or other) designation through comic text or commentary by the illustrator<br><br>Foreign name<br><br>Phenotypic representation of another ethnic group with deviations from the usual drawing style<br><br>Skin type 6<br><br>Foreign accents written out in the character's speech or thought bubble (e.g. sentence structure, grammar, spelling)	A categorization into "Recognizable as a Person of Color" was not possible based on the criteria	Person not sufficiently visible or recognizable (the person's name did not appear plus the person's head was not seen in profile or the person's face was not turned towards the viewer)<br><br>Animal character or object

Table S6: Criteria for categorizing the share of speech

Measured in whole numbers, by the number of words in the character's speech or thought bubble.<br><br>Words written together with a hyphen (e.g.: six-pack) are counted as 1 word, corresponding to compound words (e.g.: halftime).<br><br>Punctuation marks are not counted as words.<br><br>Interjections (e.g.: sigh, oh, aaah-chooo, ouch) are classified as words<br><br>A number is classified as a word.<br><br>Symbols that are pronounced (such as %, +, =, -, or €) are classified as words<br><br>Words such as "it's" (instead of "it is") are classified as 1 word
